# Regional reports for the subnational monitoring of measles elimination in Italy and the identification of local barriers to the attainment of the elimination goal

**DOI:** 10.1371/journal.pone.0205147

**Published:** 2018-10-24

**Authors:** Giovanna Adamo, Giulia Sturabotti, Valentina Baccolini, Pasquale de Soccio, Grazia Pia Prencipe, Antonino Bella, Fabio Magurano, Stefania Iannazzo, Paolo Villari, Carolina Marzuillo

**Affiliations:** 1 Department of Public Health and Infectious Diseases, Sapienza University of Rome, Rome, Italy; 2 Department of Infectious Diseases, National Institute of Health, Rome, Italy; 3 National Reference Laboratory for Measles and Rubella, National Institute of Health, Rome, Italy; 4 Infectious Diseases and International Prophylaxis Office, Ministry of Health, Rome, Italy; University of Campania, ITALY

## Abstract

Although most countries in the WHO European Region were verified in 2017 as having interrupted endemic measles transmission, nine countries were still endemic. Among these, Italy accounted for the second highest number of measles cases reported in Europe in 2017. The elimination of measles is verified at national level by each country’s National Verification Committee (NVC) through the production of an Annual Status Update (ASU). Since in Italy decentralization has led to an inhomogeneous implementation of immunization strategies among the 21 administrative Regions, the Italian NVC proposed that measles elimination should also be documented at the subnational level through regional ASUs and Synthetic Regional Reports (SRRs). The regional ASUs and the SRRs for 2014, 2015 and 2016 were produced and appraised by the NVC to evaluate the Regions’ performances in each individual year as well as over the whole period. A specific analysis of vaccination coverage, including official immunization data for 2017, was performed. Moreover, the measles epidemic of 2017 was examined. Firstly, in the period 2014–2016, low immunization rates were registered in most Regions. Sixty-three per cent of southern Regions reported rates below the national mean and an overall low-quality performance. The approval of Italy’s mandatory vaccination law in 2017 resulted in a marked increase in vaccination coverage; however, this increase was not homogeneous among Regions. Secondly, more than 50% of Regions did not report any supplemental immunization activity (SIA) for the period 2014–2016. Thirdly, from 2014 to 2016, fewer than one-third of Regions improved their reporting of outbreaks. Finally, over the study period, only two Regions reached the target required by the WHO for measles laboratory investigations. In countries with decentralized health policies, subnational monitoring can help identify local barriers to measles elimination. In Italy it has highlighted the need for further SIAs and a stronger surveillance system.

## Introduction

Although, most member states in the WHO European Region were verified in 2017 as having interrupted the endemic transmission of measles, nine countries were still endemic [1, 2]. Among these, Romania and Italy were responsible for the majority of measles cases notified in 2017 to the European Surveillance System, accounting respectively for 38% and 35% of the 14 451 cases reported [3].

In Europe as well as in most WHO Regions the verification of measles elimination is carried out by the Regional Verification Commission (RVC) based on the evidence provided by each country’s National Verification Committee (NVC) on an annual basis. A uniform documentation format–the national Annual Status Update (ASU)–is used to assist the NVC in the collection of relevant information. Special attention should be paid to monitoring the effect of any existing decentralization process on the implementation of the National Immunization Programme (NIP). In some instances, in fact, decentralization may affect the national capacity to conduct epidemiological surveillance and outbreak investigation as well as to provide complete and timely surveillance data [4].

In Italy, decentralization together with the economic crisis has led to significant disparities among the 21 Italian administrative Regions, as is particularly evident in the area of health promotion and disease prevention [5–9]. Even though the Italian NIP aims to harmonise immunization strategies between the Regions by providing a national strategy and specific objects and targets [10], Regions retain the responsibility for the implementation and management of the immunization programmes, with the consequence that these may differ from Region to Region according to their capabilities, resources and priorities [11–13]. Similarly to the NIP, the National Elimination Plan for Measles and Congenital Rubella (Piano Nazionale di Eliminazione del Morbillo e della Rosolia Congenita—PNEMoRC), which was approved in 2011 with the aim of interrupting the indigenous transmission of these two diseases by 2015 [14], establishes the general objectives and a common methodology for achieving these goals, but assigns to the Regions the task of planning and implementing at the local level the relevant operational strategies. To evaluate the progress of each Region towards the elimination of measles and rubella, in 2015 the Italian NVC proposed that the national ASU be complemented with regional ASUs and Synthetic Regional Reports (SRRs).

This paper aims to describe the results of the regional monitoring carried out through the regional ASUs and the SRRs for the period 2014–2016, highlighting progress made and barriers encountered by the Regions with specific regard to measles elimination, and the extent to which further efforts are still needed in order to reach this goal. The paper also illustrates the epidemiology of measles in Italy from 1970 to the epidemic that affected the country in 2017, and the improvements in vaccination coverage observed after the approval of the decree-law no. 73/2017 Containing Urgent Measures on the Compulsory Vaccination of Children, converted with amendments into law no. 119 of 31 July 20l7 [15].

## Methods

### National ASU and data sources

The national ASU documents and verifies the interruption of endemic measles transmission–together with that of rubella–using five core components: epidemiology, molecular epidemiology, performance of the surveillance system, population immunity and sustainability of the NIP [4].

In Italy, data on the first three core components are collected by the National Institute of Health (Istituto Superiore di Sanità, ISS) based on information reported by the Regional Health Authorities (RHAs) using a web-based surveillance system. According to this system, all suspected measles/rubella cases must be notified at national level through the ISS platform. The epidemiological investigation is performed locally while serological and/or molecular tests are carried out either by the Regional Reference Laboratories (RRLs) or the National Reference Laboratory (NRL) of the ISS [16, 17].

Data on population immunity are provided by the Ministry of Health (MoH), based on the data collected by the Regions from the respective administrative territories. The Regions are also required to collect information on any supplemental immunization activity (SIA) (i.e. strategies for delivering vaccination to children missed by routine services or to older susceptible individuals) put in place.

On an annual basis, all the above data are forwarded by the respective source to the Italian NVC, which is in charge of preparing the national ASU and submitting it to the European RVC.

### Regional ASUs and SRRs

**Fig 1:**
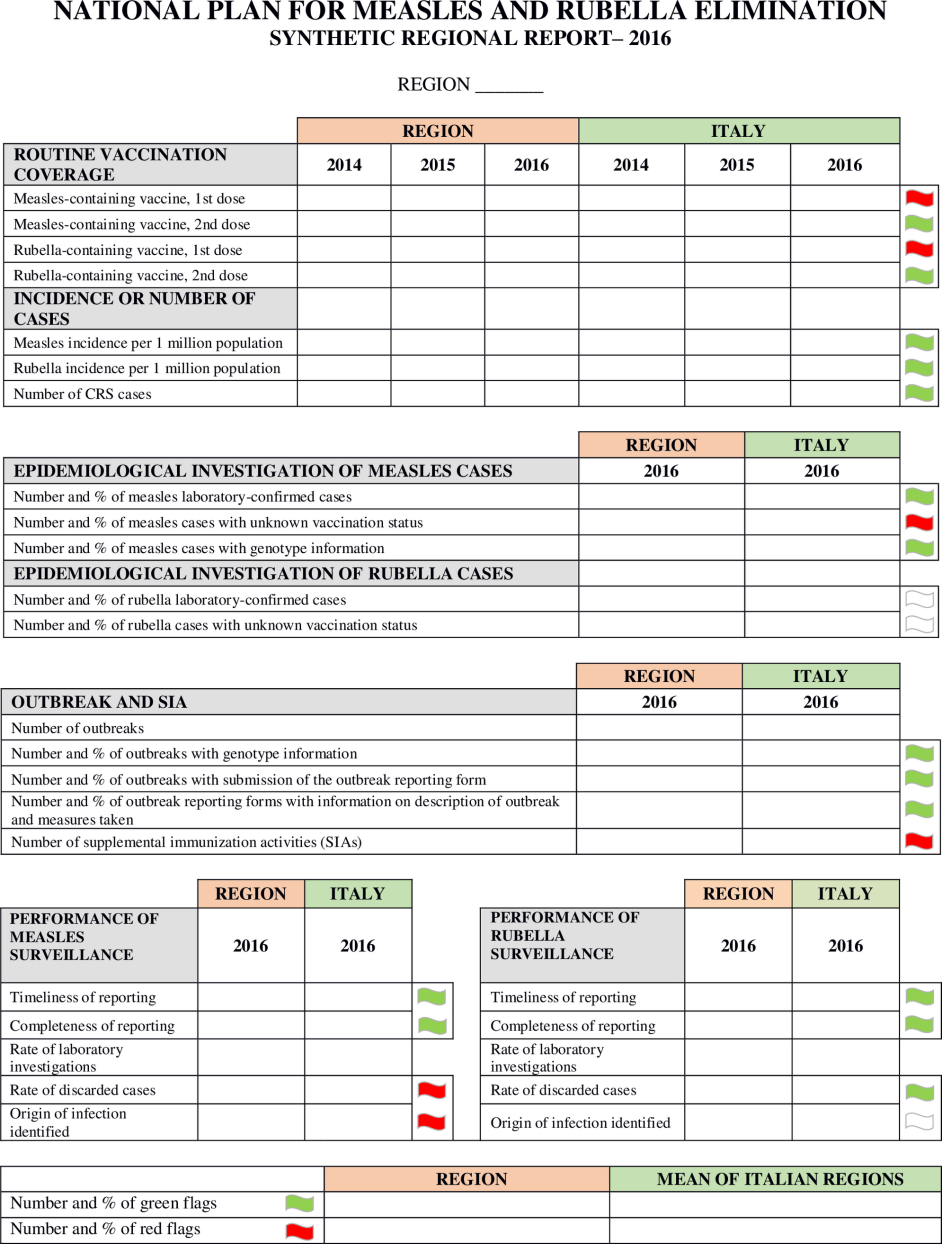
Format of the Synthetic Regional Reports (SRRs) for measles and rubella in Italy. A green or a red flag is assigned to each indicator depending on whether the regional data is, respectively, better or worse than the national mean. If the regional data cannot be evaluated, a white flag is assigned.

Data for the regional ASUs and the SRRs are obtained through the same information flow as the national ASU. The regional ASUs faithfully reproduce the structure of the national format, except that some features have been adapted for use at the subnational level. The SRRs are presented as a single page document, consisting of seven sections and a total of 27 indicators (Fig 1). The aim was to create a user-friendly tool which summarized the most important evidence from the regional ASUs, presenting it in a more intelligible way. The seven sections of the SRRs concern: i) routine vaccination coverage (four indicators); ii) incidence of measles, rubella and number of CRS cases (three indicators); iii) epidemiological investigation of measles cases (three indicators); iv) epidemiological investigation of rubella cases (two indicators); v) outbreaks and SIAs (five indicators); vi) performance of measles surveillance (five indicators); vii) performance of rubella surveillance (five indicators).

The four indicators relating to vaccination coverage, the three indicators relating to the incidence/number of cases, and the 10 indicators relating to the surveillance performance have been directly borrowed from the regional ASUs, while the remaining 10 indicators have been drawn up from scratch. For each indicator, the regional data are given together with the national mean, and compared with it using a benchmarking methodology that assigns either a green or a red flag depending on whether the regional data is, respectively, better or worse. If the regional data cannot be evaluated, a white flag is assigned. We decided to assign no flags to the rate of laboratory investigations, since this is a WHO standard indicator that considers only samples investigated in proficient laboratories, i.e. laboratories compliant with WHO standards. Since the NRL was the only laboratory meeting the WHO requirements until 2016, only the samples sent by Regions to the NRL could be considered in the calculation of this indicator. To provide a more exhaustive picture of the performance of regional laboratories that was inclusive of but not limited to the work done via the NRL, we decided to include two other indicators: the percentage of measles cases with genotype information where genotyping was performed in proficient laboratories, and the percentage of measles outbreaks with genotype information where genotyping was performed in both proficient and non-proficient laboratories. The description and meaning of the 27 indicators are presented in a table attached to each SRR (S1 Table). For some indicators, where available, WHO targets are also provided.

The regional ASUs and the SRRs for 2014, 2015 and 2016 were prepared by the NVC and shared with the 21 Regions. The production of regional ASUs and SRRs is part of a wider project funded by the Italian MoH aimed at supporting the PNEMoRC.

### Data analyses

Vaccination coverage is a key measure for evaluating the performance of the immunization system [4]. Using the SRRs produced for 2014, 2015 and 2016, we first performed a specific evaluation of regional coverage indicators, highlighting the differences between the three geographical areas of the country (North, Centre, South and Islands); official data for 2017 were also included in this step [18]. Then, in order to assess the overall regional performance, a comprehensive analysis of all indicators was conducted. Specifically, two different methods were adopted: i) comparison of the regional data with the national mean. For each year, we compared the percentage of red flags scored by each Region to the average percentage of red flags for all 21 Regions, and analysed the geographic distribution of the worst and best annual scores; for graphic purposes we assigned the red color to Regions that scored an overall percentage of red flags higher than the average percentage of red flags for all 21 Regions and the green color to Regions with a percentage score below this average; ii) evaluation of regional trends. Since the national mean score may vary from year to year, the above mentioned method is unreliable when evaluating the overall regional performance across several years. To address this issue, we analysed the trend of each indicator for the period 2014–2016, without comparing the regional data to the national average. Specifically, for indicators for which WHO targets were available, we compared the regional data to the WHO target; for indicators with no target available, we evaluated the regional trends alone.

## Results

The epidemiological situation for measles in Italy has been of particular concern in recent years, becoming particularly critical after the epidemic that affected the country in 2017. For this reason, we decided to present only results relating to the progress of the Italian Regions towards measles elimination.

### Measles in Italy from 1970 to 2017

**Fig 2:**
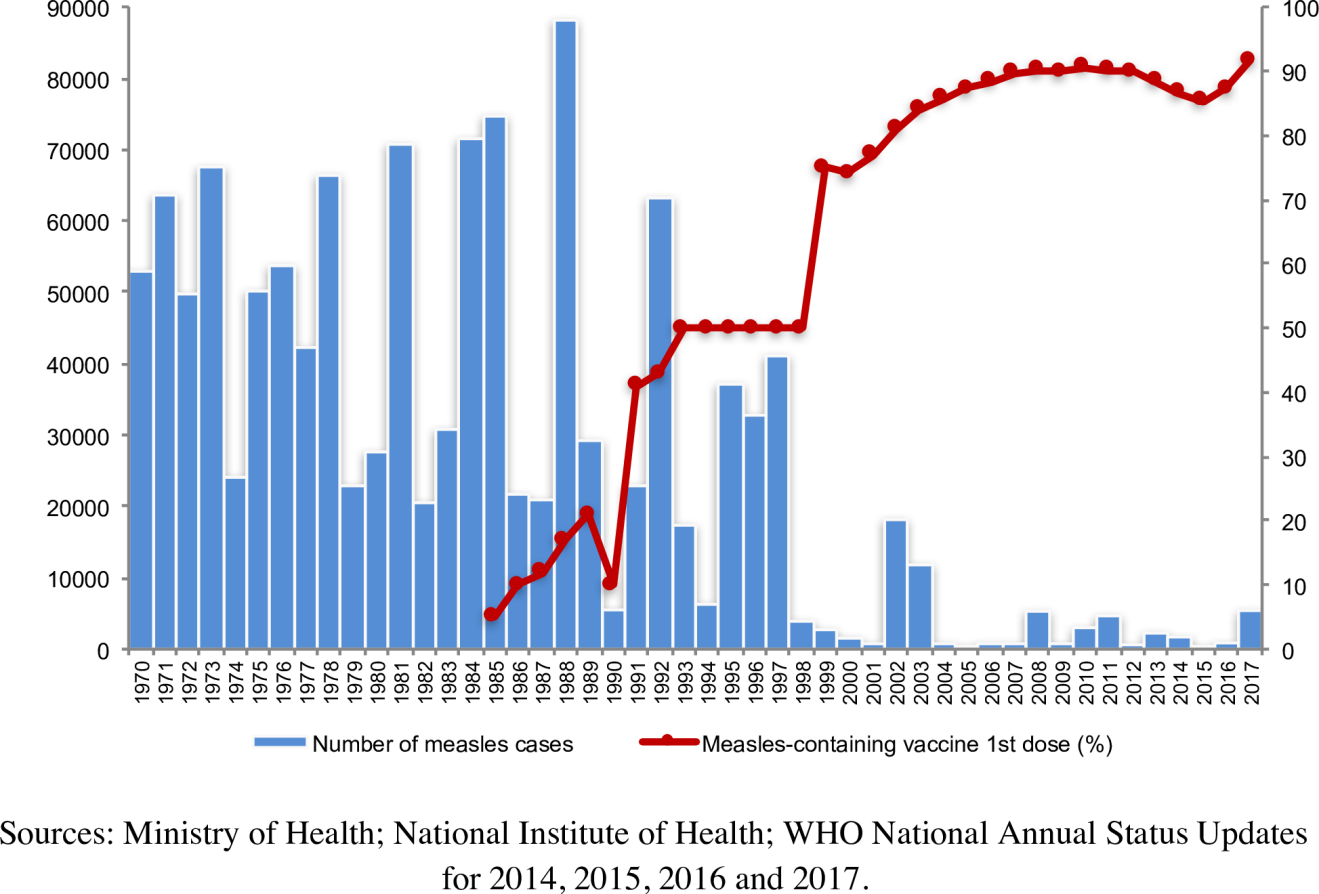
Reported measles cases and vaccination coverage for measles-containing vaccine 1^st^ dose by year, Italy, 1970–2017.

The absolute number of measles cases reported annually from 1970 to 2017 and the coverage rates for the first dose of measles-containing vaccine (MCV1) by year clearly show a general decreasing trend for measles cases and an increasing trend for vaccination coverage (Fig 2). For the first three decades, outbreaks occurred almost every two-three years, characterized by very high epidemic peaks. Since 2000, however, with the increase in vaccination coverage, there has been a dramatic reduction in the number of measles cases, along with a lengthening of the interepidemic intervals.

In 2017, after a phase of relatively low incidence during the period 2014–2016, Italy experienced a large measles epidemic with 5404 cases and four deaths. All Regions were affected but 92.0% of cases derived from just nine Regions. Eighty-seven per cent of cases were either unvaccinated or vaccinated with a single dose of MCV. Most cases (n = 3974, 73.5%) involved subjects >15 years of age, but the highest incidence occurred in children aged under one year (n = 321; 686.4 cases per million). Outbreaks mainly occurred in family and school settings, but also in hospitals, with 322 cases being reported among health care workers. Viral characterization revealed the circulation of B3, D8 and H1 genotypes during this period.

### Vaccination coverage 2014–2017

**Fig 3:**
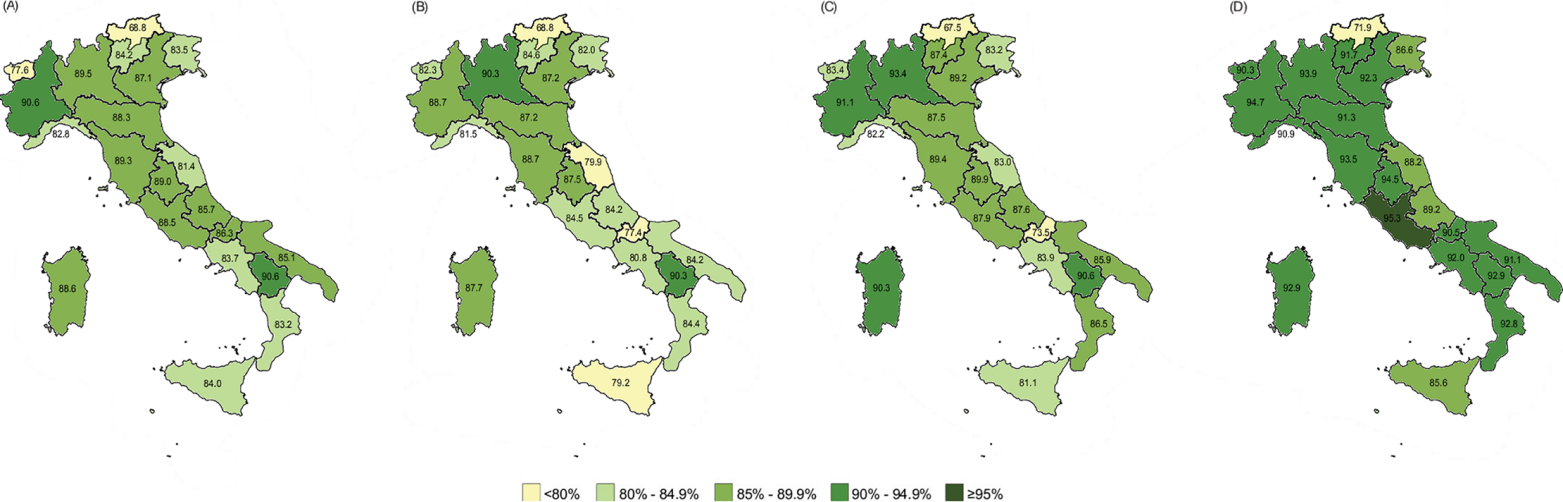
Coverage for measles-containing vaccine 1^st^ dose by Region for 2014 (A), 2015 (B), 2016 (C) and 2017 (D), Italy.

National coverage for MCV1 decreased from 86.7% in 2014 to 85.3% in 2015, and then increased to 87.3% in 2016. In this period, rates below the national average were mostly registered in the south of Italy (Fig 3A–3C). Specifically, considering the geographic distribution, in 2014 six out of eight Regions in the south (75.0%), one out of four Regions in the centre (25.0%) and five out of nine Regions in the north (55.6%) reported rates below the national average. A similar situation was observed in 2015, with six out of eight Regions in the south (75.0%), two out of four Regions in the centre (50.0%) and five out of nine Regions in the north (55.6%) having reported rates below the national average. In 2016, five out of eight Regions in the south (62.5%), four out of nine Regions in the north (44.4%) and one out of four Regions in the centre (25.0%) were below the national mean. Official data for 2017 show that national coverage increased by 4.4 percentage points (i.e. to 91.7%) compared to 2016. However, eleven Regions out of 21 reported rates below the national data (Fig 3D): four out of eight Regions in the south (50.0%), one out of four Regions in the centre (25.0%) and six out of nine Regions in the north (66.7%).

**Fig 4:**
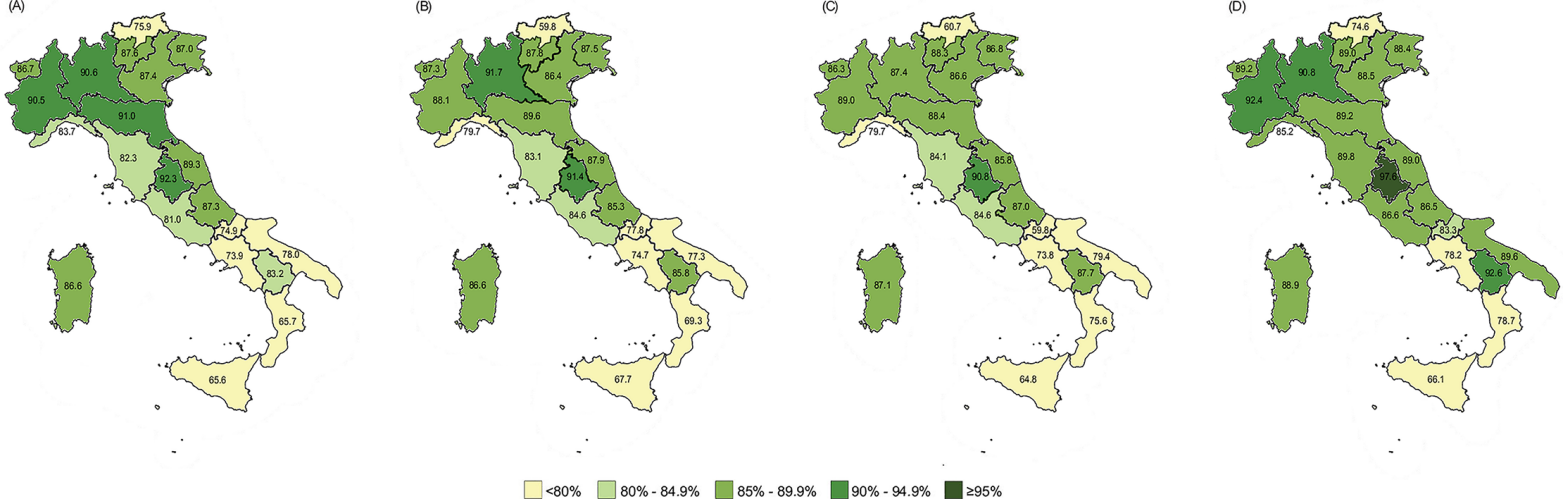
Coverage for measles-containing vaccine 2^nd^ dose by Region for 2014 (A), 2015 (B), 2016 (C) and 2017 (D), Italy.

National coverage for the second dose of MCV (MCV2) stagnated at around 83% from 2014 to 2016 (82.7% in 2014; 83% in 2015; 82.2% in 2016). The lowest rates were again registered in the south of the country (Fig 4A–4C), with five out of eight southern Regions (62.5%) reporting vaccination coverage below the national average in all three years under review. In 2017, national coverage increased to 85.8% (Fig 4D). Four out of eight Regions in the south (50.0%) and two out of nine Regions in the north (22.2%) reported rates below the national average.

### Performance of the Italian Regions in the elimination process

#### Comparison of the regional data with the national mean

**Fig 5:**
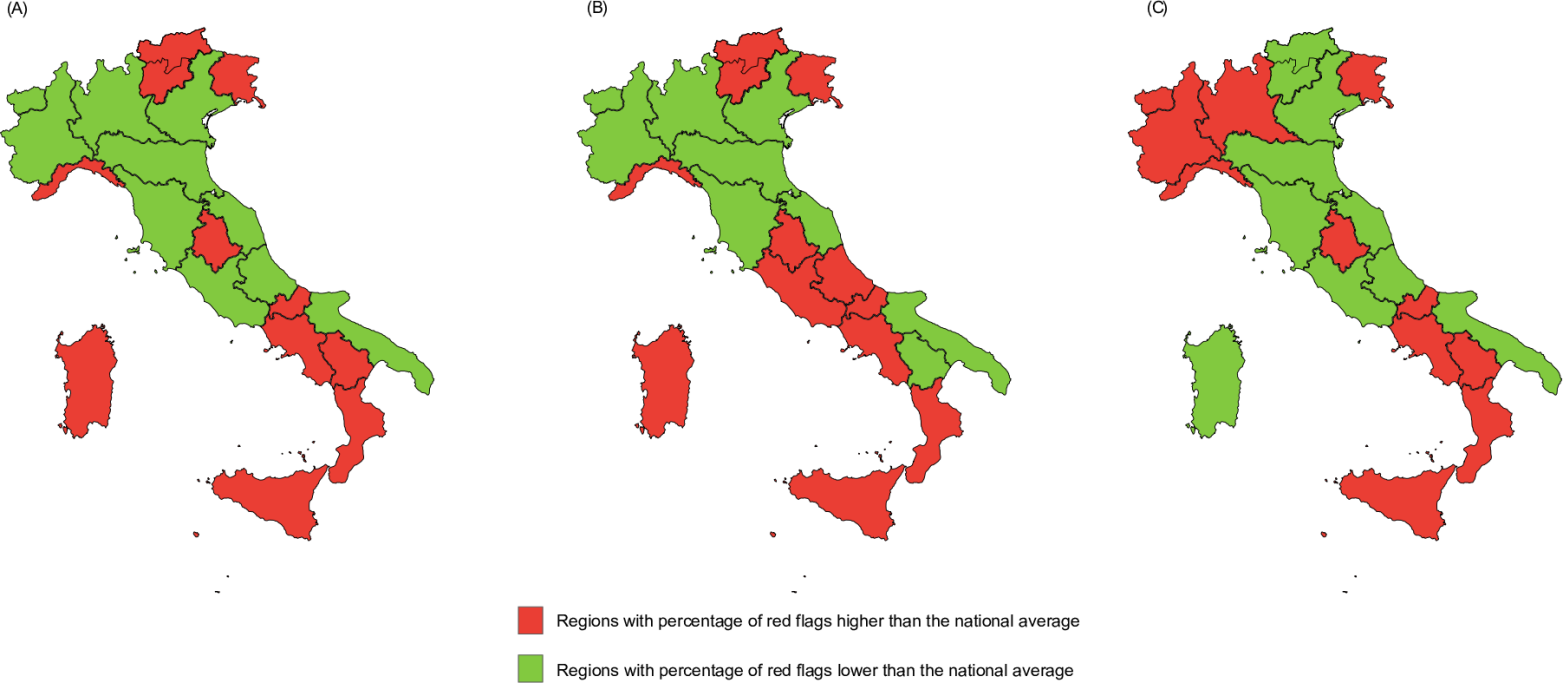
Scores of the Synthetic Regional Reports for 2014 (A), 2015 (B) and 2016 (C), Italy.

The annual analysis of the SRRs showed that most Regions in the south of Italy performed worse than the national average in all three years under review (Fig 5). Specifically, looking at the geographic distribution of the “red” scores, six out of eight southern Regions (75.0%), one out of four central Regions (25.0%) and four out of nine northern Regions (44.4%) were “red” in 2014. Similarly, six out of eight southern Regions (75.0%), two out of four central Regions (50.0%) and four out of nine northern Regions (44.4%) were “red” in 2015, while five out of eight southern Regions (62.5%), one out of four central Regions (25.0%), and five out of nine northern Regions (55.6%) were “red” in 2016.

### Evaluation of regional trends

Regional trends for the 27 SRR indicators were evaluated for the period 2014–2016 (Table 1). Based on the results obtained, Regions were allocated into three categories: well-performing Regions conforming to WHO targets; improving Regions; and Regions with no data available (see Table 1 for exact definitions). For the purposes of this article, we highlight only results relating to measles.

**Table 1:** Results of the trend analysis for the 27 indicators of the synthetic regional reports for the period 2014–2016, and evaluation of target achievements.

**Indicator**	WHO Target	Well-performing Regions* N (%)	Improving Regions** N (%)	Regions with no data available*** N (%)
**Routine vaccination coverage**				
**Measles-containing vaccine. 1st dose^a^**	*≥95%*	0 (0.0)	11 (52.4)	0 (0.0)
**Measles-containing vaccine. 2nd dose^a^**	*≥95%*	0 (0.0)	7 (33.3)	0 (0.0)
**Rubella-containing vaccine. 1st dose^a^**	*≥95%*	0 (0.0)	11 (52.4)	0 (0.0)
**Rubella-containing vaccine. 2nd dose^a^**	*≥95%*	0 (0.0)	7 (33.3)	0 (0.0)
**Incidence or number of cases**				
**Measles incidence per 1 million population^b^**	*<1 case / 1*,*000*,*000*	3 (14.3)	11 (52.4)	0 (0.0)
**Rubella incidence per 1 million population^b^**	*<1 case / 1*,*000*,*000*	17 (81.0)	2 (9.5)	0 (0.0)
**Number of CRS cases^c^**	*<1 case / 100*,*000*	20 (95.2)	0 (0.0)	0 (0.0)
**Epidemiological investigation of measles cases**				
**Percentage of lab-confirmed cases**			9 (42.9)	3 (14.3)
**Percentage of cases with unknown vaccination status^d^**			11 (52.4)	3 (14.3)
**Percentage of measles cases with genotype information**			4 (19.0)	11 (52.4)
**Epidemiological investigation of rubella cases**				
**Percentage of lab-confirmed cases**			1 (4.8)	13 (61.9)
**Percentage of cases with unknown vaccination status^d^**			6 (28.6)	13 (61.9)
**Outbreaks and Supplemental Immunization Activities (SIAs)**				
**Number of outbreaks^e^**			15 (71.4)	0 (0.0)
**Percentage of outbreaks with genotype information**			10 (47.6)	6 (28.6)
**Percentage of outbreaks with submission of the outbreak reporting form**			5 (23.8)	6 (28.6)
**Percentage of outbreak reporting forms with description of outbreak and measures taken**			6 (28.6)	6 (28.6)
**Number of SIAs^f^**			5 (23.8)	0 (0.0)
**Performance of measles surveillance**				
**Timeliness of reporting**	*≥80%*	17 (81.0)	2 (9.5)	0 (0.0)
**Completeness of reporting**	*≥80%*	17 (81.0)	2 (9.5)	0 (0.0)
**Rate of laboratory investigations**	*≥80%*	2 (9.5)	3 (14.3)	1 (4.8)
**Rate of discarded cases^g^**	*≥2 discarded cases /* *100*,*000 population*	0 (0.0)	2 (9.5)	0 (0.0)
**Origin of infection identified**	*≥80%*	14 (66.7)	0 (0.0)	3 (14.3)
**Performance of rubella surveillance**				
**Timeliness of reporting**	*≥80%*	17 (81.0)	2 (9.5)	0 (0.0)
**Completeness of reporting**	*≥80%*	17 (81.0)	2 (9.5)	0 (0.0)
**Rate of laboratory investigations**	*≥80%*	4 (19.0)	1 (4.8)	7 (33.3)
**Rate of discarded cases^g^**	*≥2 discarded cases /* *100*,*000 population*	0 (0.0)	3 (14.3)	0 (0.0)
**Origin of infection identified**	*≥80%*	7 (33.3)	0 (0.0)	11 (52.4)
**Total**		21 (100)	21 (100)	21 (100)

Notes

* (only for indicators with WHO targets): Regions that have either maintained for the period considered, or achieved at the end of that period, the relative WHO target.

**: Regions that have improved over the period considered in accordance with the definition of improvement given to each indicator (see notes a-g).

***: Regions with no data available, i.e. Regions for which the indicator in question could not be evaluated due to the unavailability of data for either two or all of the three years considered.

a: Improvement is defined as a ≥0.5 percentage point increase in vaccination coverage in the last year available, compared to the first year.

b: Improvement is defined as a decrease of ≥1 case per 1 million population in the incidence of measles/rubella in the last year available, compared to the first year.

c: Improvement is defined as a decrease of ≥1 case in the number of CRS cases notified in the last year available, compared to the first year.

d: Improvement is defined as a ≥1 percentage point decrease in the percentage of cases with unknown vaccination status in the last year available, compared to the first year; Regions are also considered to be improving if 0 cases with unknown vaccination status were reported in both years.

e: Improvement is defined as a ≥1-point reduction in the number of outbreaks notified in the last year available, compared to the first year; Regions are also considered to be improving if 0 outbreaks were reported in both years.

f: Improvement is defined as a ≥1-point increase in the number of SIAs reported in the last year available, compared to the first year.

g: Improvement is defined as a ≥0.2 percentage point increase in the rate of discarded cases in the last year available, compared to the first year.

For all remaining indicators, improvement is defined as a ≥1 (percentage) point increase in the rate/percentage registered in the last year available, compared to the first year.

The analysis of regional trends showed that none of the Italian Regions achieved the target of ≥95% for either MCV1 or MCV2 in the period 2014–2016. Eleven out of 21 Regions (52.4%) improved with regard to MCV1; seven Regions (33.3%) improved with regard to MCV2, while only five Regions (23.8%) improved with regard to both MCV1 and MCV2 over the three years. In 2017 two Regions achieved the target of ≥95% (Lazio for MCV1 and Umbria for MCV2 in 2017) (Figs 3D and 4D).

A downward trend in the incidence of measles was reported by 11 out of 21 Regions (52.4%) in the period 2014–2016. Overall, three Regions (14.3%) reached the target of <1 case per one million population. However, underreporting must be considered while assessing these data.

Results for the epidemiological investigation of measles cases reveal a contrasting picture in regional laboratory performances. While data for the first two indicators relating to diagnostic accuracy (percentage of lab-confirmed cases and percentage of cases with unknown vaccination status) showed an improvement in approximately half of the Regions (42.9% and 52.4%, respectively), an improvement was observed in only four Regions (19%) for the indicator relating to genotyping. However, as stated previously, only genotyping performed in laboratories compliant with WHO standards has been considered in the calculation of this indicator. The Italian NRL was the only laboratory meeting WHO standards until 2016. Therefore, we were only able to calculate this indicator for those Regions that sent their samples to the NRL.

The data on section outbreaks and SIAs are rather heterogeneous. While results for the first two indicators (number of outbreaks and percentage of outbreaks with genotype information) appear fairly good, with the latter in particular revealing how the performance of the Regions improves when samples investigated by regional laboratories are also included, results for the other indicators highlight some critical issues with the Italian surveillance system and the PNEMoRC. The third and fourth indicators refer to the outbreak reporting system. According to the WHO guidelines, an outbreak reporting form should be filled every time an outbreak occurs [19]. However, the use of such forms is not yet part of the Italian web-based surveillance system; RHAs provide them as ex-post documentation requested by the MoH in order to complete the ASU, with the consequence that most of the information relating to the epidemiological investigation conducted and the control measures taken may be inadequately collected or even lost in retrospect. Results show, in fact, that less than one-third of the Regions improved with regard to these two indicators (23.8% and 28.6%, respectively). Data on the number of SIAs performed are of great concern: only five Regions (23.8%) registered an improving trend, while more than half of the Regions (52.4%) reported zero SIAs for all three years.

The surveillance performance of the Italian Regions has been evaluated using some of the WHO standard indicators of the National ASU, which have been adapted for use at the regional level in both the regional ASUs and the SRRs. The results for measles surveillance show varying trends for the different indicators. For the first two indicators, 17 Regions out of 21 (81%) achieved the WHO target of ≥80%, but for laboratory investigations, only two Regions (9.5%) achieved the WHO target. We must point out, however, that this indicator could only be calculated for those Regions that sent their samples to the NRL. The rate of discarded cases, which refers to the number of suspected cases investigated and discarded as non-measles using laboratory testing in a proficient laboratory and/or epidemiological linkage to another confirmed disease, is used to measure the sensitivity of measles surveillance [20]. This is a critical indicator of Italian surveillance performance, as demonstrated by the fact that none of the Regions reached the WHO target in the period 2014–2016, while almost all Regions (19/21, 90.5%) reported <0.2 cases/100 000. Results for the last indicator showed a good performance in two thirds of the Regions (14/21, 66.7%) with regard to the identification of the origin of measles viruses.

## Discussion

Despite much evidence supporting prevention as the most-cost effective way to maintain health in a sustainable manner [21–23], concerns about upfront costs and the intangibility of outcomes too often lead governments, particularly during periods of economic crisis, to spend only a small fraction of health budgets on promoting health and preventing disease [24]. Decentralization–a phenomenon relevant to Italy as well as other European countries–is another factor that could undermine prevention activities. In Italy, regionalization has led to significant differences among regional health services, and this is particularly evident in the field of prevention [5–9]. There is much evidence that Regions with a financial deficit, which are subject to Recovery Plans (RPs) that aim to restore economic stability, tend to invest mainly in short term interventions with a high impact on health care costs, at the expense of health promotion and disease prevention, erroneously considered to have an impact only in the longer run [5, 6, 25].

The results of our regional analyses clearly show that this is also true in the area of measles elimination where six out of the seven Regions that were under a RP during the study period (Abruzzo, Molise, Campania, Puglia, Calabria and Sicilia) did poorly. Among these, four Regions in particular (Molise, Campania, Calabria and Sicilia) reported vaccination coverage rates below the national mean for both MCV1 and MCV2 in all years considered. These same Regions were also always “red” in the SRRs annual analyses, having performed worse than the national average for all three years under review.

Monitoring the elimination process not only at national but also at regional level can therefore contribute to the identification of regional differences in the implementation of the PNEMoRC that can act as barriers to measles elimination [26–28]. The analysis of regional performances through the SRRs highlighted in particular four aspects that, in our opinion, can hinder the achievement of Italy’s elimination goals and should be primarily addressed. The first of these aspects, likely to be the most important, relates to the low immunization rates registered in most of the Italian Regions in the period 2014–2016. Vaccine hesitancy is regarded as one of the leading causes of the decline in vaccination coverage in recent years [29–36]. To address this issue, in June 2017 the Italian parliament approved the decree-law no. 73/2017 Containing Urgent Measures on the Compulsory Vaccination of Children, converted with amendments into law no. 119 of 31 July 20l7, which makes vaccination compulsory for measles and rubella, increasing the number of mandatory vaccinations from four to 10, for children from 0 to 16 years of age [15]. Official results provided for 2017 by the MoH show an increase of 4.4 percentage points for MCV1 and of 3.6 for MCV2, compared to 2016 [18]. However, the increase in routine vaccination coverage has not been homogeneous among Italian Regions. The persistence of different organizational models in the offer of vaccination underlines the importance of monitoring the advancements at subnational level to ensure a uniform implementation of vaccination policies across the country.

The second aspect is that, although the new mandatory law should improve immunization rates for children and young people up to the age of 16, additional immunization activities should be implemented for older birth cohorts and other susceptible groups [37, 38]. Indeed, official immunization data for 2017 reveal that coverage rates for subjects of 18 years of age are still below 85% [18]. If it is true that in 2017 Regions have been mostly committed to enacting the measures provided for by the new mandatory law, consequently devoting less time and resources to the implementation of SIAs, it is also true that SIAs have also been implemented infrequently in the previous years (2014–2016).

Thirdly, public health institutions should also prioritize the strengthening of the outbreak reporting system. Our analysis showed that less than one-third of Regions improved outbreak reporting over the study period. A possible solution to this problem is to integrate the WHO outbreak reporting form into the web-based surveillance platform, so that this form is filled as an outbreak occurs, rather than retrospectively.

Finally, the presence of a strong laboratory surveillance system is essential for documenting the interruption of endemic transmission, and this needs to be further strengthened in Italy. Our analysis showed poor results in a number of Regions with regard to molecular investigation. The establishment in 2017 of a subnational laboratory network (MoRoNet), which encompasses 12 laboratories in 11 Regions, supervised by the NRL and compliant with WHO MR LabNet standards [39], has already strengthened the collaboration between these two levels, making it possible to investigate 83.2% of measles cases in proficient laboratories and to genotype 61.5% of outbreaks in 2017.

The regional monitoring through regional ASUs and SRRs was mentioned by the WHO as one of the activities put in place in Italy to support the epidemiological analysis and the identification of weaknesses and strengths in regional elimination plans [40]. To our knowledge, this is the first attempt to systematically evaluate the status of measles elimination at the subnational level in a country of the WHO European Region. Despite this, our study has some limitations that must be acknowledged. First, the comparison of the regional data with the national mean could be misleading, since there is a concrete possibility that an indicator with a green flag, albeit better than the national mean, is well below the WHO target. Second, in the evaluation of regional trends the definition of improving Regions for each indicator was made on the grounds of cut-off values that were arbitrarily chosen. However, these comparisons and the evaluation of the regional trends were made only for descriptive purposes, to give Regions a general idea of their performance compared to the rest of the country and over time. In any case, WHO targets, where available, were always specified.

## Conclusions

In countries with decentralized health policies, monitoring progress towards elimination goals at both national and subnational levels can help to harmonize the implementation of the Elimination Plan and ensure its long-term sustainability. The experience within the Italian context proves that regional ASUs and SRRs can be useful in identifying barriers to the attainment of elimination goals at subnational level and in finding operational solutions to help overcome such barriers. At the regional level these reports could also be used as a simple and easy-to-understand tool to obtain the commitment of both technicians and politicians, both of which play an important role in the achievement of the elimination goals.

## Supporting information

S1 Table Description and meaning of the 27 indicators that make up the Synthetic Regional Reports. (PDF)
