# Supplementary material for: Regional reports for the subnational monitoring of measles elimination in Italy and the identification of local barriers to the attainment of the elimination goal
Source: PLoS One. 2018 Oct 24;13(10):e0205147. doi: 10.1371/journal.pone.0205147 (PMC6200208; doi:10.1371/journal.pone.0205147)
Supplement: S1 Table — (PDF) [file pone.0205147.s001.pdf]

S1 Table. Description and meaning of the 27 indicators that make up the Synthetic Regional Reports.

For some indicators, where available, WHO targets are also provided.

INDICATOR	DESCRIPTION	MEANING, TARGETS AND NOTES
**ROUTINE VACCINATION COVERAGE**		
**Measles/Rubella-containing vaccine, 1st dose**	Number of children <=24 months of age with one dose of measles/rubella containing vaccine given after 12 months of age<br>―――――<br>Number of children 12-24 months of age resident in the Region	-If the regional data is equal or higher than the national mean (for the year 2016), a green flag is assigned. If the regional data is lower than the national mean, a red flag is assigned.<br>**-The elimination target required by the WHO is >=95%**
**Measles/Rubella-containing vaccine, 2nd dose**	Number of children that will make their 7th birthday in the year for which the vaccine coverage is calculated, with the second dose of measles/rubella containing vaccine given<br>―――――<br>Number of children resident that will make their 7th birthday in the year for which the vaccine coverage is calculated	- If the regional data is equal or higher than the national mean (for the year 2016), a green flag is assigned. If the regional data is lower than the national mean, a red flag is assigned.<br>**-The elimination target required by the WHO is >=95%**
**INCIDENCE**		
**Measles/Rubella incidence per 1 million population**	Total number* of measles/rubella cases<br>―――――<br>Region's population<br><br>* excluding imported cases	-If the regional data is equal or lower than the national mean (for the year 2016), a green flag is assigned. If the regional data is higher than the national mean, a red flag is assigned.<br>**-The elimination target is < 1 case per million population during the last 12 months**
**Number of CRS cases**	Total number of CRS cases in the Region	-If the regional data is equal or lower than the national mean (for the year 2016), a green flag is assigned. If the regional data is higher than the national mean, a red flag is assigned. The national mean is obtained by dividing the total number of CRS cases reported in the country by the number of Regions (21).<br>**-The elimination target is < 1 case per 100,000 live births**

INDICATOR	DESCRIPTION	MEANING, TARGETS AND NOTES
**EPIDEMIOLOGICAL INVESTIGATION OF MEASLES/RUBELLA CASES**		
**Number and % of measles/rubella laboratory-confirmed cases**	Number of laboratory-confirmed measles/rubella cases<br>――――――――――<br>Total number* of measles/rubella cases<br><br>* including imported cases	-If the regional data is equal or higher than the national mean, a green flag is assigned. If the regional data is lower than the national mean, a red flag is assigned. If the regional data cannot be evaluated (both numerator and denominator are = 0), a white flag is assigned.
**Number and % of measles/rubella cases with unknown vaccination status**	Number of measles/rubella cases with unknown vaccination status<br>――――――――――<br>Total number* of measles/rubella cases<br><br>* excluding imported cases	-If the regional data is equal or lower than the national mean, a green flag is assigned. If the regional data is higher than the national mean, a red flag is assigned. If the regional data cannot be evaluated (both numerator and denominator are = 0), a white flag is assigned.
**Number and % of measles cases with genotype information**	Number of measles cases with genotype information<br>――――――――――<br>Total number of measles cases tested in a WHO-accredited or proficient laboratory with a positive test result	-If the regional data is equal or higher than the national mean, a green flag is assigned. If the regional data is lower than the national mean, a red flag is assigned. If the regional data cannot be evaluated (both numerator and denominator are = 0), a white flag is assigned.<br>-The Italian National Reference Laboratory (NRL) of the National Institute of Health was the only laboratory meeting WHO standards until 2016. Therefore this indicator has been calculated only for those Regions that have sent their samples to the NRL.

INDICATOR	DESCRIPTION	MEANING, TARGETS AND NOTES
**OUTBREAK AND SIA**		
**Number of outbreaks**	Number of outbreaks in the Region	-No flags are assigned to this indicator.
**Number and % of outbreaks with genotype information**	Number of outbreaks with genotype information / Total number of outbreaks in the Region	-If the regional data is equal or higher than the national mean, a green flag is assigned. If the regional data is lower than the national mean, a red flag is assigned. If no outbreaks have been reported by the Region, a white flag is assigned.
**Number and % of outbreaks with submission of the outbreak reporting form**	Number of outbreaks with submission of the outbreak reporting form / Total number of outbreaks in the Region	-If the regional data is equal or higher than the national mean, a green flag is assigned. If the regional data is lower than the national mean, a red flag is assigned. If no outbreaks have been reported by the Region, a white flag is assigned.
**Number and % of outbreak reporting forms with information on description of outbreak and measures taken**	Number outbreak reporting forms with information on description of outbreak and measures taken / Total number of outbreaks in the Region	-If the regional data is equal or higher than the national mean, a green flag is assigned. If the regional data is lower than the national mean, a red flag is assigned. If no outbreaks have been reported by the Region, a white flag is assigned.
**Number of supplemental immunization activities (SIAs)**	Number of supplemental immunization activities conducted in the Region	-If at least one SIA has been conducted in the Region, a green flag is assigned. If no SIAs have been implemented, a red flag is assigned.

INDICATOR	DESCRIPTION	MEANING, TARGETS AND NOTES
**PERFORMANCE OF MEASLES/RUBELLA SURVEILLANCE**		
**Timeliness of reporting**	Number of reports submitted by the Region to the National Institute of Health by the deadline<br>———<br>Number of expected reports (12)<br><br>*including "zero" reports	-If the regional data is equal or higher than the national mean, a green flag is assigned. If the regional data is lower than the national mean, a red flag is assigned.<br>**-The target required by the WHO is >=80%**
**Completeness of reporting**	Number of reports submitted by the Region to the National Institute of Health<br>———<br>Number of expected reports (12)<br><br>* including "zero" reports	-If the regional data is equal or higher than the national mean, a green flag is assigned. If the regional data is lower than the national mean, a red flag is assigned.<br>**- The target required by the WHO is >=80%**
**Rate of laboratory investigations**	Number of suspected measles/rubella cases with adequate specimens collected and tested in a WHO-accredited or proficient laboratory<br>———<br>Number of suspected* measles/rubella cases<br><br>* including laboratory-confirmed, clinically compatible and discarded cases but excluding epidemiologically linked cases	-No flags are assigned to this indicator, since, until 2016, in most of the Regions laboratory investigations have been conducted in non-accredited laboratories.<br>**-The target required by the WHO is >=80%.**
**Rate of discarded cases**	Number of suspected measles/rubella cases investigated and discarded as non-measles or non-rubella cases<br>———<br>Region's population	-If the regional data is equal or higher than the national mean, a green flag is assigned. If the regional data is lower than the national mean, a red flag is assigned.<br>**-The target required by the WHO is at least 2 discarded measles/rubella cases per 100,000.**
**Origin of infection identified**	Number of measles/rubella cases for which the origin of infection (e.g. imported, import-related or endemic) has been identified<br>———<br>The total number of measles or rubella cases<br><br>* including imported cases	-If the regional data is equal or higher than the national mean, a green flag is assigned. If the regional data is lower than the national mean, a red flag is assigned. If the regional data cannot be evaluated (both numerator and denominator are = 0), a white flag is assigned.<br>**-The target required by the WHO is >=80%.**
